# Evaluation of a pipeline for simulation, reconstruction, and classification in ultrasound-aided diffuse optical tomography of breast tumors

**DOI:** 10.1117/1.JBO.27.3.036003

**Published:** 2022-03-24

**Authors:** Giuseppe Di Sciacca, Giulia Maffeis, Andrea Farina, Alberto Dalla Mora, Antonio Pifferi, Paola Taroni, Simon Arridge

**Affiliations:** a University College London, Department of Computer Science, London, United Kingdom; b Politecnico di Milano, Dipartimento di Fisica, Milano, Italy; c Istituto di Fotonica e Nanotecnologie, Consiglio Nazionale delle Ricerche, Milano, Italy

**Keywords:** diffuse optical tomography, ultrasound, lesion classification, breast cancer, breast digital phantom

## Abstract

**Significance:**

Diffuse optical tomography is an ill-posed problem. Combination with ultrasound can improve the results of diffuse optical tomography applied to the diagnosis of breast cancer and allow for classification of lesions.

**Aim:**

To provide a simulation pipeline for the assessment of reconstruction and classification methods for diffuse optical tomography with concurrent ultrasound information.

**Approach:**

A set of breast digital phantoms with benign and malignant lesions was simulated building on the software VICTRE. Acoustic and optical properties were assigned to the phantoms for the generation of B-mode images and optical data. A reconstruction algorithm based on a two-region nonlinear fitting and incorporating the ultrasound information was tested. Machine learning classification methods were applied to the reconstructed values to discriminate lesions into benign and malignant after reconstruction.

**Results:**

The approach allowed us to generate realistic US and optical data and to test a two-region reconstruction method for a large number of realistic simulations. When information is extracted from ultrasound images, at least 75% of lesions are correctly classified. With ideal two-region separation, the accuracy is higher than 80%.

**Conclusions:**

A pipeline for the generation of realistic ultrasound and diffuse optics data was implemented. Machine learning methods applied to a optical reconstruction with a nonlinear optical model and morphological information permit to discriminate malignant lesions from benign ones.

## 1 Introduction

Diffuse optical tomography (DOT) is a technique of medical imaging based on the injection and collection of near-infrared and visible red photons undergoing scattering and absorption events in tissues.^1^^,^^2^ The evolution of the related technology has recently seen the miniaturization of the electronic components involved, thus leading to the design of handheld probes.^3^^–^^5^ However, the mathematical reconstruction problem stemming from DOT is severely ill-posed and reconstructed images are low resolution and low contrast, and present artifacts, which hinders the wide use of the technique in clinical scenarios.

Nonetheless, DOT presents qualities that make it an active and growing research topic, e.g., the spectral nature of DOT, which allows functional analysis of tissues and is crucial for clinical diagnosis. Other qualities are its noninvasiveness and cost-effectiveness.^6^^–^^10^ Among its applications, DOT is currently utilized for breast cancer diagnosis.^11^^,^^12^ In the field, x-ray mammography is often referred to as the state of the art for breast cancer screening, but it presents decreased sensitivity with dense breasts. In such cases, ultrasound (US) imaging is often used as an adjunct technique. The diagnosis via US B-mode images is done at a morphological level and allows the clinician to reach a sensitivity close to 100%.^13^ With DOT, US shares the noninvasiveness and cost-effectiveness, but it is also characterized by higher resolution. For these reasons, the combination of DOT with a well-posed, high-resolution techniques such as US imaging was proposed early in the field^14^^,^^15^ together with the question on how to best achieve an improvement of DOT out of this combination in the wider topic of multimodal imaging.^16^^–^^19^

In general, the inverse problem in DOT is solved via the minimization of an objective function in the following form:

$$f^{\mathrm{recon}}=\mathrm{argmin}\;D(Af,g)+\alpha \Psi (f),$$ (1)

where D is a—usually quadratic—data-fitting term, A is the forward operator, g is the data, Ψ is a regularizer, and α is the hyperparameter weighting the regularization with respect to the data-fitting term. The forward model of propagation A is chosen based on, e.g., linearity, time dependency, domain discretization. Many approaches have been proposed in literature for the combination of DOT with other techniques: US information can be incorporated either in the regularizer^18^^,^^20^^,^^21^ or directly in the model.^22^^–^^25^ Often, the technical specifications of a probe and the main objective of a study define the best reconstruction strategy to adopt, but newly designed physical devices require a time span of months or even years before being assessed on clinically relevant cases and extract statistical figures.^26^^,^^27^ Having realistic simulations would, in some degree, help assessing proposed reconstruction approaches before clinical evaluation also from a statistical point of view. Recently, VICTRE was proposed as a tool for tomosynthesis of realistic breast digital phantoms for applications in z-ray mammography^28^ and was subsequently adopted as a test database for other optical imaging applications.^29^^–^^31^ Hereby, we propose a simulation pipeline based on VICTRE for the generation of functional phantoms for a combined US+DOT hand held probe, adopting—as an example—the integrated probe developed in the SOLUS Project.^32^ Based on this, we identify a reconstruction method and a classification procedure with the aim of best discriminating benign lesions from malignant ones. To our knowledge, it is the first time that US and DOT standalone simulations are combined for the characterization of such a multimodal probe. A strategy for the combination of acoustic and optical properties of tissues has instead already been presented in Refs. 30 and 33. In Secs. 2.1 and 2.2, we show the procedure used to generate respectively US simulations and optical data from a VICTRE digital phantom. In Secs. 2.2.1 and 2.3, we present the reconstruction method that has been adopted to retrieve the optical properties of the simulated lesions and the classification method to separate them into benign and malignant.

## 2 Methods

VICTRE is a simulation pipeline aiming at giving a realistic tomosynthesis of breast digital phantoms.^28^^,^^31^ A digital phantom is a voxelized image, where each voxel is assigned to tissue class: glandular, adipose, artery, vein, terminal duct lobular unit, duct, nipple, skin, muscle, and ligament.^30^^,^^34^ For simplicity, in the following discussion, we refer to a VICTRE phantom as an operator V:Ω→N assigning an index i representing a tissue to a region in 3D space so i=Vr. The effect is a decomposition of the domain Ω in subsets $\Omega_{i}$ composed of a single tissue so $\Omega =\cup_{i}\Omega_{i}$. The software also allows for the compression that the breast undergoes in a standard mammography exam and for the generation of tumor-like shapes.^35^ We simulated a total of $N_{\mathrm{ph}}∼700$ left breasts where the fat-fraction was the main parameter that was changed to simulate the most frequent types of breasts. The resolution was set to 0.5 mm as a compromise between the resolution of US and computational effort.

Here, we choose to compress the breast to 45 mm along the axis going from head to feet to simulate the mild compression given by a handheld probe with flat surface. We note that this arrangement does not take into account some peculiarities of handheld devices such as the effects of the chest wall. For each digital phantom, a lesion of average radius varying from 6 to 13.5 mm was generated and inserted in the tissues after compression.

**Fig. 1:**
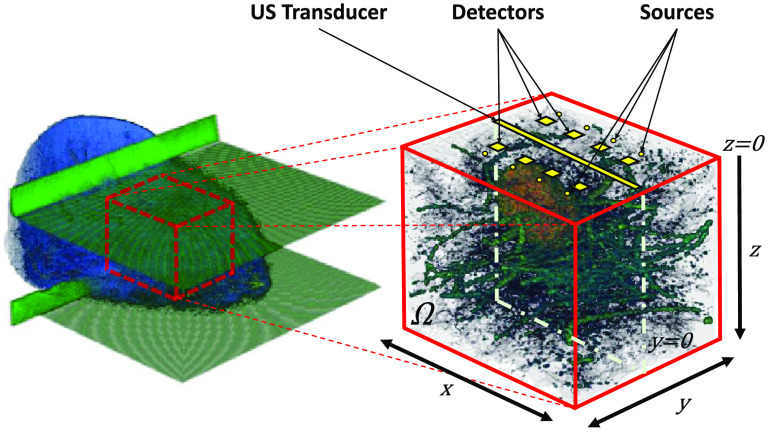
Geometry definition. A VICTRE phantom is compressed between two paddles to 45 mm. A cuboid region Ω is extracted to serve as functional ground truth in the discussion. A SOLUS-like probe is simulated on the top surface of the considered domain. The plane y=0 is set to be the imaging plane of the US transducer.

Building on VICTRE, we present here a simulation pipeline for a dual-mode probe inspired by the multimodal device developed in the EC funded SOLUS Project.^5^ A whole acquisition is composed by a B-mode image set along the plane y=0 and by a set of time diffuse optical data obtained in reflectance geometry by eight sources and eight detectors placed on the plane z=0, identifying the top surface of the breast. The center of the US transducer and of the matrix of detectors is set to be at the origin of the reference system. We show a schematic of the simulated acquisition system in Fig. 1. In detail, the sources are disposed on two rows of four elements each at a distance of 17.5 mm from and parallel to the US imaging plane. The distance between each source on the same line is 12 mm. An analog geometry is selected for the detectors, but at a distance 10.8 mm from the plane y=0.

For simplicity, each lesion was placed in the phantom so as to have its center of mass at a maximum distance of 0.3l from the US imaging plane y=0, with l the maximum elongation of the lesion along y. In the following, we present a simulation approach to generate US and optical data from the VICTRE phantoms. In Sec. 2.2.1, we present an optical reconstruction method based on a two-region nonlinear model and, in Sec. 2.3, we apply some commonly used classification methods to assess the separability of the reconstructed lesions into benign and malignant classes. The whole procedure of generation and compression of the digital phantoms took circa 3 h on a virtual machine of 20 GB of RAM assembled on a computational cluster.

### 2.1 US Modeling

Computational US is an ever-growing branch of research since field-II made its first appearance in 1996.^36^ Subsequently, many US simulators have been proposed and each of them differs from its competitors in a variety of factors such as the model of propagation for acoustic waves, the numerical method used or the physical model for tissues. For this work, we considered as simulators Field-II,^36^ SIMUS,^37^ CREANUIS,^38^ and k-wave.^39^ A thorough comparison of these software is out of scope in this paper, thus we summarize here the main differences on methodology and aims. Apart from k-Wave, all the considered simulators have a mesh-free numerical solver: acoustic scatterers with defined properties and positions are placed in the computational domain and an acoustic field is then propagated. The signal recorded at the transducer is the result of the interaction of the acoustic field with the scatterers. This approach allows a certain flexibility in simulating the position and translation of scatterers in tissues. Speckles in the simulated B-mode images arise from the random position of scatterers, while the general acoustic properties of the medium are usually kept constant.

Field-II is based on the concept of a transducer’s impulse response function, the main assumptions being: a linear wave equation, scatterers acting as monopole sources and weak scattering phenomena. By the Huygens’ principle, in a homogeneous medium the spatial impulse response function $h(r_{1},t)$ at $r_{1}$ from an aperture surface S is:^40^

$$h(r_{1},t)=\int_{S}\frac{\delta (t-\frac{\left|r_{1}-r_{2}\right|}{v_{s}})}{2\pi |r_{1}-r_{2}|}dS,$$ (2)

where δ(t) is a point-like source, $r_{2}$ is the position of the transducer, and $v_{s}$ is the speed of sound in the medium. Thus, the emitted pressure field can be retrieved by the convolution: $p(r_{1},t)=\rho_{0}\frac{\partial }{\partial t}|u|{*}_{r}h(r_{1},t)$ with ${*}_{r}$ spatial convolution operator, thus getting the Rayleigh integral,^36^ with $\rho_{0}$ density of the medium and u is the velocity of the front face of the transducer. In this scenario, a point-like change in pressure and sound speed in the medium can be introduced by a term $f_{m}(r_{1})$ so that:

$$p(r_{1},t)=v_{pe}(t){*}_{t}f_{m}(r_{1}){*}_{r}h_{pe}(r_{1},t),$$ (3)

where $v_{pe}$ is the electronic impulse response function, $h_{pe}$ is a two-ways impulse response function taking into account the transmitting and receiving impulses, and ${*}_{t}$ denotes a convolution in time. Equation (3) is solved for a number of scatterers placed in the medium, and the results are summed to generate one B-mode scan line. We refer to Refs. 41 and 42 for details. The simulation assumptions used in SIMUS are the same as Field-II.^37^ The main computation is performed in Fourier space and it is fully open source. This brings a set of numerical advantages and ease of use, especially for what regards frequency-dependent characteristics of ultrasound imaging. However, as Field-II, SIMUS is limited to linear propagation of acoustic waves and to single scattering phenomena that do not allow to reproduce, artifacts such as reverberation, shadowing, or mirror image artifacts. The addition of a heterogeneous nonlinear parameter and a GPU-based solver are the main features brought by CREANUIS.^38^^,^^43^^,^^44^ The software is still particle-based, however, under the approximation of weak nonlinear phenomena, two pressure fields are propagated, one that is linear $p^{\left(1\right)}$ and a second nonlinear one $p^{\left(2\right)}$:

$$(\nabla^{2}-\frac{1}{v_{s}}\frac{\partial }{\partial t})p^{\left(1\right)}=0,$$ (4)

$$(\nabla^{2}-\frac{1}{v_{s}^{2}}\frac{\partial }{\partial t})p^{\left(2\right)}=-(1+\frac{B}{2A})\frac{1}{p_{0}v_{s}^{4}}\frac{\partial^{2}}{\partial t^{2}}p^{(1)2},$$ (5)

where $v_{s}$ is the speed of sound in the medium, $p_{0}$ is the background pressure, and $\frac{B}{A}$ is the nonlinearity parameter. The propagation of the two fields is made in Fourier domain and solved via the GASM method.^44^ The second harmonic field is dependent on the value of the fundamental one. As with Field-II and SIMUS, the scan lines are obtained by placing scattering particles in the medium and backpropagating both fields to the transducer. The main limitations of the particle-based simulators described above is the small flexibility in simulating a heterogeneous speed of sound, density, and other spatial properties in tissues, that are only handled by the position and amplitude of scatterers. In turns, this limits the number of real-like artifacts that can be obtained from a simulation.

k-Wave is a mesh-based nonlinear simulator based on the set of acoustic equations:^39^^,^^45^^,^^46^

$$\left\{ \begin{array}{l} \frac{\partial }{\partial t}u=-\frac{1}{\rho_{0}}\nabla p\\ \frac{\partial }{\partial t}\rho =(2\rho -\rho_{0})\nabla \cdot u-u\cdot \nabla \rho_{0}\\ p=v_{s}^{2}(\rho +\frac{B}{2A}\frac{\rho^{2}}{\rho_{0}}+d\cdot \rho_{0}-L\rho ) \end{array},\right.$$ (6)

where $\rho_{0}$ is the background mass density of the medium, ρ, p, and u are the acoustic mass density, pressure, and particle speed, respectively. L is a fractional Laplacian term accounting for dispersion and acoustic absorption. Nonlinear acoustic effects are governed by the terms 2ρ and $\frac{B}{2A}\rho^{2}$ with $\frac{B}{A}$ nonlinearity parameter.^47^ Acoustic sources are simulated by adding a source term to the first and/or second equation. The set of equations are solved with a pseudospectral method with the spatial components of the acoustic quantities that are calculated in k-space. One B-mode scan-line is obtained by defining a grid, a source, a detector, a medium and solving the set of Eq. (6) in the system. In principle, the approach followed in k-Wave allows one to simulate heterogeneous media and artifacts such as speckles, shadowing, and reverberations that come naturally from the wave equation and allow for more realistic simulations.^48^

A schematic comparison of the considered US simulators, modeled after the one in Ref. 48 can be found in Table 1. Given its characteristics, k-Wave has been chosen as the main tool for US simulations in this paper.

**Table 1:** Summary of the properties of the considered simulators.

	Field-II	SIMUS	CREANUIS	k-Wave
Numerical method	Meshfree	Meshfree	Meshfree	Mesh-based
Governing acoustic equations	Linear	Linear	Non-linear	Non-linear
Time domain	Temporal	Harmonic	Temporal	Both^48^
Space domain	x,y,z	x,y,z	$k_{x},k_{y},k_{z}$	x,y,z+k
Medium	Homo.	Homo.	Hete.	Hete.
Scattering	Weak	Weak	Weak	Multiple
Artifacts	No	No	No	Yes

#### 2.1.1 Definition of the medium

The system in Eq. (6) describes the contrast in a US image as the result of spatially changing acoustic properties—speed and density—in tissues.^49^ Two different scales of contrast are observed in a B-mode breast image: a macroscopic one, visible at the interfaces of tissues and a microscopic one, that is due to changes in characteristic impedance at the cellular level and results in regions of different brightness in the images depending on the tissues.^50^ We present a VICTRE-based acoustic digital phantom that aims at having the same dual scale of heterogeneities. To each tissue identified by the index i=Vr is assigned an average sound speed and standard deviation such that for each point r in $\Omega_{i}$ the speed of sound $v_{s}(r)$ for $r\in \Omega_{i}$ is defined to be a stationary Gaussian process GP such that

$${\overset{¯}{v}}_{s,i}∼N(\mu_{i},\sigma_{i,\text{macro}})\;{\left\{v_{s}\right\}}_{r\in \Omega_{i}}∼\mathrm{GP}({\overset{¯}{v}}_{s,i},\sigma_{i,\text{micro}}),$$ (7)

where N(μ,σ) is a normal distribution of mean μ and standard deviation σ, ${\overset{¯}{v}}_{si}$ defines the average speed of sound in the tissue i, and it is extracted from a normal distribution of mean $\mu_{i}$ and standard deviation of $\sigma_{i,\text{macro}}$. The standard deviation $\sigma_{i,\text{micro}}$ acts as a source of microscopic contrast depending on the tissues. The mean speed of sound μ and macroscopic standard deviation $\sigma_{\text{macro}}$ of adipose tissues, glandular tissues, and lesions have been assigned following Ref. 51. In Figs. 4(a) and 4(b), we give an example of simulated maps for ${\overset{¯}{v}}_{s}$ and the piecewise function $\sigma_{\text{micro}}$ over the imaging plane y=0 of Ω. The resulting speed of sound can be found in Fig. 4(c). The acoustic properties of the remaining tissues were drawn from the same distribution as the glandular ones. The microscopic contrast in the lesions was selected to be lower than the level of noise of all the surrounding tissues, as often happens in US B-mode images where lesions are less bright. The microscopic standard deviation for each of the other tissues was randomly selected to have a standard deviation between 1% and 5% of the average speed of sound of the tissues. To reduce the computational cost of the simulations, only the active elements (42 out of 256 in total) of the whole transducer are simulated at the same time; for each scan line the smaller transducer is moved along the scanning direction. Thus, it is possible to simulate a portion of the simulated medium for each scan line, so it is possible to have a finer grid allowing to simulate higher US frequencies.^39^ With these settings, a B-mode image took ∼7  h to run on a virtual machine with a 10 GB GPU assembled on a computational cluster. In Table 2, we give the main simulation parameters used. Where possible the simulations parameter have been set to be equal to those of the final SOLUS probe. Even though some speckles are present in the images, we note that to model fully developed speckles, a high number of scatterers per wavelength would be required. We choose here to limit the number of scatterers to reduce the computational effort.

**Table 2:** Main simulation parameters for ultrasound B-mode images.

$f_{c}$	7 MHz
Focus	15 mm
Elevation plane focal point	16 mm
Fractional bandwidth	80%
N elements	256
Height	4 mm
Pitch	0.2 mm
Kerf	0.001 mm
Width	0.198 mm
Scan lines	200
Grid size point	516×172×86
Grid size	45×15×7.5 mm
Δx,Δy,Δz	0.0872 mm
Perfectly matched layer	30×10×5
Δt	8.9 ns
T	67 μs
$v_{s,0}$	1465 m/s

### 2.2 Optical Modeling

Photon propagation of monochromatic light in tissues is well described by the radiative transfer equation:^52^

(1c∂∂t+s^·∇+μa+μs)Lν(r,s^,t)=μs∫Ω^′Lν(r,s^,t)Θ(s^·s^′)dΩ^′+Q(r,s^,t), (8)

where $L_{\nu }$ is the spectral radiance, Q is a source term, s^ is the direction of propagation, Θ is a phase function, and $\mu_{s}$ and $\mu_{a}$ are the scattering and absorption coefficients. Even though some analytical solutions for simple systems are known, the RTE can be effectively solved only with Monte Carlo methods. A valid alternative to the RTE in human tissues is given by the diffusion equation (DE) with Robin boundary conditions:^53^

{1c∂∂tΦ(r,t)−∇·κ∇Φ(r,t)+μaΦ(r,t)=Q(r,t)  in  Ω×[0,T],Φ(r,t)+2Aκ∂∂n^Φ(r,t)=0  on  ∂Ω, (9)

where photons migration is described via a diffusive process of the fluence Φ in a time T and is dependent on the properties of absorption $\mu_{a}$ and diffusivity $\kappa =\frac{1}{3{\mu_{s}}^{\prime }}$ of the medium,^52^^,^^54^^–^^57^ where ${\mu_{s}}^{\prime }$ is the reduced scattering coefficient. Here, $A=\frac{1+R}{1-R}$ with R refraction coefficient. The DE can be analytically solved for simple geometries and homogeneous optical coefficients. The solution of the DE for complex systems and heterogeneous coefficients requires the use of numerical solvers such as the finite element method (FEM).^58^ In general, the forward model to calculate the signal observed by a detector in position $r_{j}$ due to a source i can be expressed as a forward operator:

$$y_{i,j}≔\Phi_{i}(r_{j},t)=A_{i,j}[\begin{array}{c} \mu_{a}(r)\\ {\mu_{s}}^{\prime }(r) \end{array}].$$ (10)

Experimental conditions are incorporated in the model by convolving the exitance with the experimental impulse response function measured with a time-resolved diffuse spectroscopy laboratory research prototype.^59^ Among the many numerical tools available for the implementation of FEM, we choose the software TOAST++.^60^

The optical coefficients $\mu_{a}$ and ${\mu_{s}}^{\prime }$ can be linked to the functional properties of the tissues via a spectral model:^61^^–^^63^

$$\mu_{a}(r,\lambda )=\underset{i}{\sum }\varepsilon_{i}(\lambda )C_{i}(r),$$ (11)

$${\mu_{s}}^{\prime }(s,\lambda )=a(r){\left(\frac{\lambda }{\lambda_{0}}\right)}^{-b(r)}.$$ (12)

**Fig. 2:**
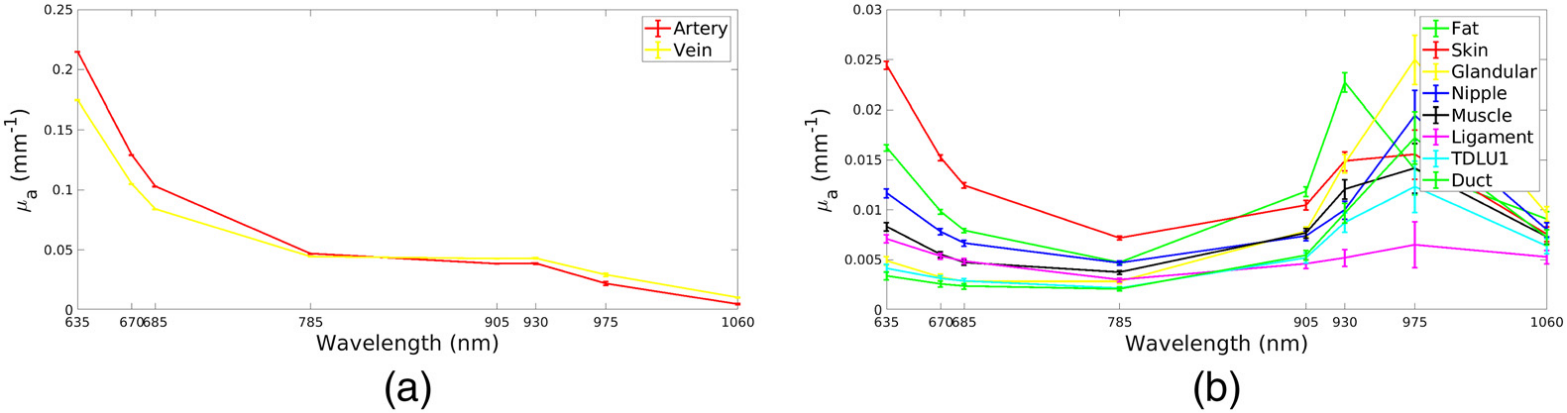
Spectra used in simulations for the components generated by VICTRE. Blood vessels and other tissues are presented in different images for visualization purposes due to the scale. (a) Absorption spectra of blood vessels. A smaller saturation has been chosen for veins. (b) Absorption spectra simulated for the other tissues.

These expressions define a spectral model that relates the functional properties of tissues to a set of $2\times N_{\lambda }$ optical coefficients, where $N_{\lambda }$ is the number of the probing wavelengths. We use the functional information of each VICTRE phantom to assign an optical ground truth to each tissue. The wavelengths chosen for the simulations of the system are 635, 670, 685, 785, 905, 930, 975, 1060 nm as explored in Ref. 64. This set of wavelengths is suitable for the retrieval of a number of chromophores: hemoglobin, oxyhemoglobin, lipids, water, and collagen. For each tissue present in the digital phantoms, a chromophore concentration was drawn and then inserted in Eq. (11) to retrieve its absorption spectrum. In Fig. 2, we show the simulated absorption spectra for each tissue.

The coefficients a were drawn from a normal distribution such that $a∼N(1.5\;{\mathrm{mm}}^{-1},0.25\;{\mathrm{mm}}^{-1})$ for benign lesions and $a∼N(1.4\;{\mathrm{mm}}^{-1},0.25\;{\mathrm{mm}}^{-1})$ for malignant ones. In addition, for each λ a term $\eta ∼N(0,0.1{\mu_{s}}^{\prime }(\lambda ))$ was added to ${\mu_{s}}^{\prime }(\lambda )$ as a source of noise in the model in Eq. (11).

**Fig. 3:**
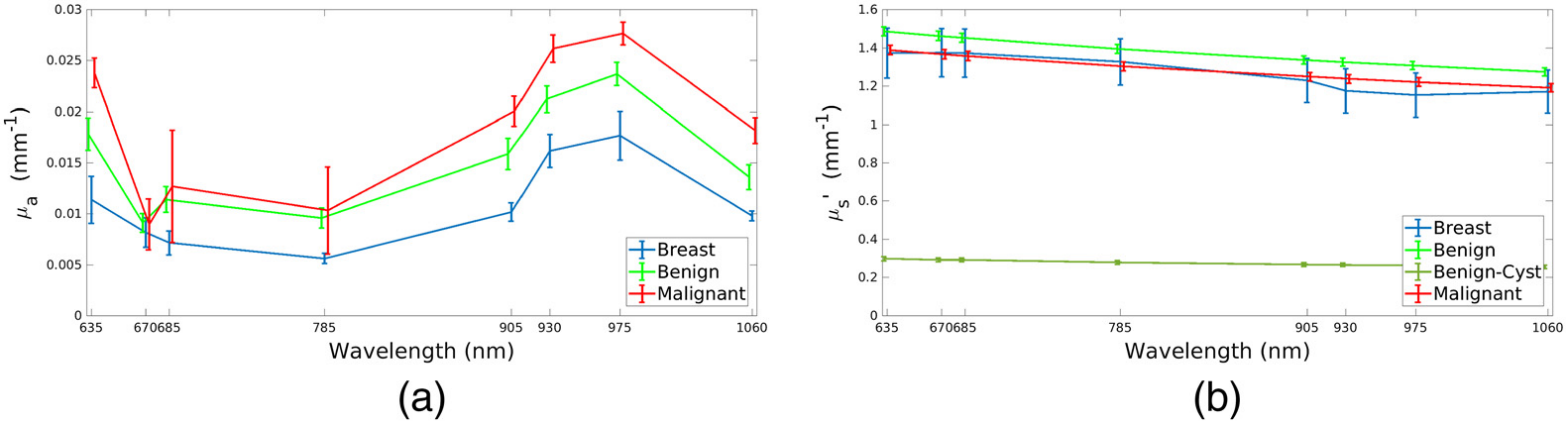
Spectral plot of optical coefficients for breast bulk (blue), benign (green), and malignant (red) inclusions. The effective optical coefficient values for the breast have been obtained by fitting a homogeneous analytical model to the reference optical data obtained by simulating a breast with no inclusion. (a) Absorption spectra of malignant lesions, benign lesions, and healthy breast tissues. (b) Scattering spectra of malignant lesions, benign lesions (excluding cysts), cysts, and healthy breast tissues.

A set of optical data was simulated without any lesion. A nonlinear global fit of the homogeneous optical coefficients $\mu_{a}$ and ${\mu_{s}}^{\prime }$ was then applied.^65^ The average reconstructed optical coefficients are shown in Fig. 3.

For the optical properties of breast lesions and their connection to benign and malignant nature, we refer to Ref. 9 to assign their optical properties. To take into account also cysts, in the simulations 25% of benign lesions were set to have $a∼N(0.3\;{\mathrm{mm}}^{-1},0.01\;{\mathrm{mm}}^{-1})$. The number of benign and malignant lesions was 349 and 379, respectively. No spatial variation of the optical coefficients inside each tissue was simulated at this stage. Unless specified otherwise, we refer to the simulated optical properties of lesions as $\mu_{a}^{\text{truth}}$ and $\mu_{s}^{\prime \text{truth}}$ (i.e., ground truth). In Fig. 3, we display the set of the absorption and scattering properties simulated for benign lesions, malignant lesions and the effective optical properties for breast healthy tissues. The latter have been obtained generating optical data from the breast without inclusion and fitting an homogeneous analytical optical model to the data.

Optical data were simulated with a cuboid domain of computation of side 64×58×30  mm with cubic voxels of side 2 mm. Time stepping was performed by an implicit Eulerian scheme with a time step of 25 ps for a total of 400 steps. Sources were defined to be of the Neumann type, with Gaussian profile of width 0.5 mm at t=0 and to be zero for t>0. Detectors were defined to have a Gaussian profile of width 1 mm. Noise was modeled by sampling a Poisson variable with mean and variance equal to an expected number of detected photons $N^{\text{expect}}$. This number was estimated as the number of photons detectable at the largest source–detector distance for a 1-min clinical examination with large area SIPM detectors^66^^,^^67^ and is equal to the area under the curve, i.e., $N^{\text{expect}}=\int_{-\infty }^{\infty }y_{i,j}dt$. The photon counts for other source–detector distances were rescaled linearly by their individual integrals over time. We note that, as a result of the compression, the dimensions of the phantoms are on average 6.3% smaller than the domain of computation for the optical data. For ease of computation, the part occupied by air was assigned to adipose or glandular tissue depending on which tissue was prevalent in the remainder of the breast.

#### 2.2.1 Reconstruction

For the optical reconstruction, we choose a two-region nonlinear diffusion forward model in the form:

$$y_{i,j}=[A_{i,j}[h]](t+t_{\text{shift}})\;\text{with }h≔[\begin{array}{c} \mu_{a}^{\text{in}}\\ \mu_{a}^{\text{bulk}}\\ {\mu_{s}}^{\prime \text{in}}\\ {\mu_{s}}^{\prime \text{bulk}} \end{array}],$$ (13)

where $\mu_{a}^{\mathrm{in}}$ and ${\mu_{s}}^{\prime \text{in}}$ are the coefficients inside the inclusion, $\mu_{a}^{\text{bulk}}$, ${\mu_{s}}^{\prime \text{bulk}}$ are the optical coefficients in the bulk, and $t_{\text{shift}}$ is a fictitious time shift parameter that is included to improve convergence in reconstruction. The operator A is the same as Eq. (10) and the operator χ applied to h returns the optical coefficients over the whole domain. χ supposes prior knowledge on the morphology of the domain, e.g., by means of ultrasound information.

As with the forward model used in simulations, we include the instrumental impulse response function to the forward model as a convolution. To limit nonidealities present in any experimental system such as noise and amplitude mismatches, we test here an approach based on automatic selection of a region of interest (ROI), binning and self-normalization of data. For each source–detector couple, the preprocessed data results in a curve of $N_{\mathrm{TW}}$ temporal steps and area 1. Here, we automatically select a temporal ROI ranging from $T_{1}$ where the curve first reaches the 0.1 of its peak to $T_{N_{\mathrm{TW}}}$ where it last reaches values of 0.01 of the peak. A set of $N_{\mathrm{TW}}=80$ equally spaced bins $\left(T_{k},T_{k+1}\right)$ for $k=1,\cdots ,N_{\mathrm{TW}}$ along the ROI were selected. We can define a preprocessing operator

$$P_{k}[y(t)]:=\frac{\int_{T_{k}}^{T_{k+1}}y(t)dt}{\overset{N_{\mathrm{TW}}}{\underset{k=1}{\sum }}\int_{T_{k}}^{T_{k+1}}y(t)dt},$$ (14)

which extracts the k’th bin from the self-normalized measurements.

Reconstructions for each wavelength are performed by minimization of an objective function of the form:

$$L(h)=\frac{1}{2}\overset{N_{Q}}{\underset{i=1}{\sum }}\overset{M_{D}}{\underset{j=1}{\sum }}\overset{N_{\mathrm{TW}}}{\underset{k=1}{\sum }}\frac{{\left(P_{k}[y_{i,j}(t)]-P_{k}[A_{i,j}[\chi h](t+t_{\mathrm{shift}})]\right)}^{2}}{\sigma_{i,j,k}^{2}(t)dt},$$ (15)

where $\sigma_{i,j,k}^{2}=\frac{P_{k}[y_{i,j}(t)]}{\overset{N_{\mathrm{TW}}}{\underset{k=1}{\sum }}\int_{T_{k}}^{T_{k+1}}y_{i,j}(t)dt}$ under the assumption of Poisson noise. We minimize the objective function by means of the MATLAB function lsqcurvefit with constraints on the values of h so $0<\mu_{a}<0.06\;{\mathrm{mm}}^{-1}$ and $0<{\mu_{s}}^{\prime }<2.2\;{\mathrm{mm}}^{-1}$.^68^ The absolute values of the fictitious time parameter were limited to 25 ps. Only source–detector distances bigger than 27 mm have been considered. Thus, a subset of all the available curves was selected to allow for convergence of the absorption parameters over scattering^52^ as well as to mitigate the effect of local perturbations, e.g., because of highly absorbing blood vessels that hinder the validity of a two-region model. The fitting procedure took circa 60 min per wavelength for each phantom on a virtual machine with 300 MB of GPU. Overall, the procedure of simulation and reconstruction took circa 18 h per breast, with the most important hardware requirements needed for the simulation of B-scan mode images.

### 2.3 Classification

We investigate the feasibility of machine learning classification methods to the reconstructed optical properties $\mu_{a}^{\text{in}}$ and $\mu_{s}^{\prime \text{in}}$ of the inclusions for a total of 16 features per lesion. To ease the visualization of 16-dimensional data, a dimensionality reduction with principal components analysis (PCA) was employed. As a result of this preliminary analysis, log-normalization of data was applied to guarantee a higher degree of separability. Among the many techniques that are available in literature, three main methods have been used for classification: logistic regression, support vector machines (SVM), and a fully connected network.^69^ A nonlinear SVM was implemented with the Python library scikit-learn.^70^ The SVM was optimized by choosing which kernel amongst Gaussian, polynomial, and sigmoidal and what regularization parameter C ranging from 0.5 and 5 guaranteed the minimum accuracy error on the training dataset. Separation of the dataset for logistic regression and SVM was done with a split ratio of 60:40 between training and test datasets. A fully connected neural network (FCN) was implemented with the Python library Tensorflow^71^ and is composed of four layers with ReLU activation function for the hidden layers and a sigmoid for the final layer. The width of the fully connected layers were 16, 32, 16, and 1, respectively. Hinge-loss was selected as loss function.^72^ Separation of the dataset was done with a split ratio of 60:20:20 between training validation and test datasets. Quantification of the performances of the mentioned techniques was performed by means of accuracy (acc.), precision (prec.), recall (rec.), and F1-score (F1) defined as

$$\mathrm{acc}.=\frac{\mathrm{TM}+\mathrm{TB}}{\mathrm{TB}+\mathrm{TM}+\mathrm{FB}+\mathrm{FM}}\;\mathrm{prec}.=\frac{\mathrm{TM}}{\mathrm{TM}+\mathrm{FM}}\;\mathrm{rec}.=\frac{\mathrm{TM}}{\mathrm{TM}+\mathrm{FB}}\;F1=\frac{2\mathrm{prec}.\times \mathrm{rec}.}{\mathrm{acc}.+\mathrm{prec}.},$$ (16)

where TM is the number of correctly identified malignant lesions, TB is the number of correctly benign ones, while FM and FB are the numbers of incorrectly identified malignant and benign lesions, respectively.

## 3 Results

We assess our results from three points of view. First, we qualitatively show the simulated US B-mode images. After that, we apply the prior extraction procedure as described in Ref. 73. As a second assessment, we analyze the performances of the optical reconstructions. As a last step, we show how the reconstruction impacts on the separability of the lesions into benign and malignant classes.

### 3.1 Assessment of US Simulations and Distance Transform

**Fig. 4:**
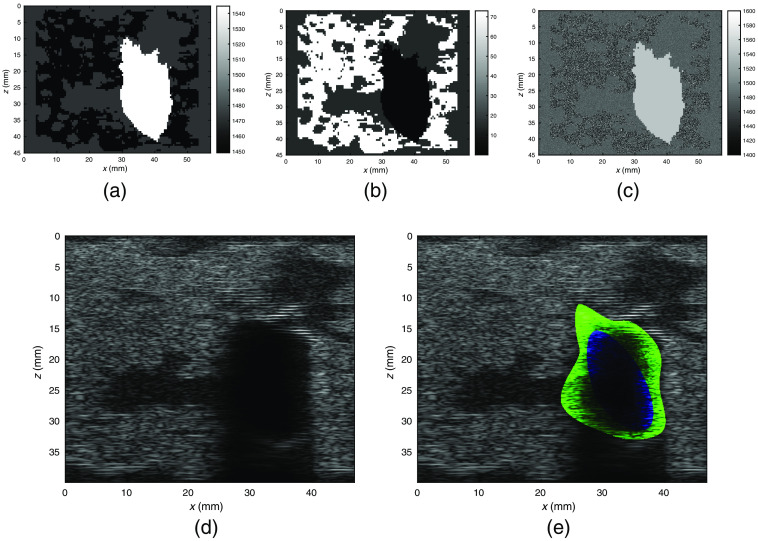
B-mode image generation: Example 1. (a) Map of ${\bar{v}}_{s}$ over ${\Omega |}_{y=0}$. (b) Map of $\sigma_{\text{micro}}$ over ${\Omega |}_{y=0}$. (c) $v_{s}(r)$ for $r\in {\Omega |}_{y=0}$. (d) B-mode image. (e) Segmentation. Blue is the user-defined segmentation, green is the final one. SDI=0.80, dA=−0.26.

**Fig. 5:**
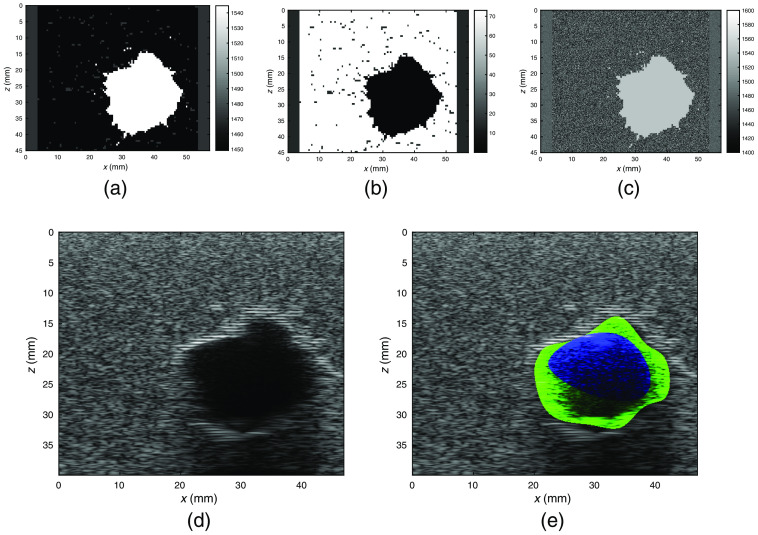
B-mode image generation: Example 2. (a) Map of ${\overset{¯}{v}}_{s}$ over $\Omega {|}_{y=0}$. (b) Map of $\sigma_{\mathrm{micro}}$ over $\Omega {|}_{y=0}$. (c) $v_{s}(r)$ for $r\in \Omega {|}_{y=0}$. (d) B-mode image. (e) Segmentation. Blue is the user-defined segmentation, green is the final one. SDI=0.76, dA=−0.32.

**Fig. 6:**
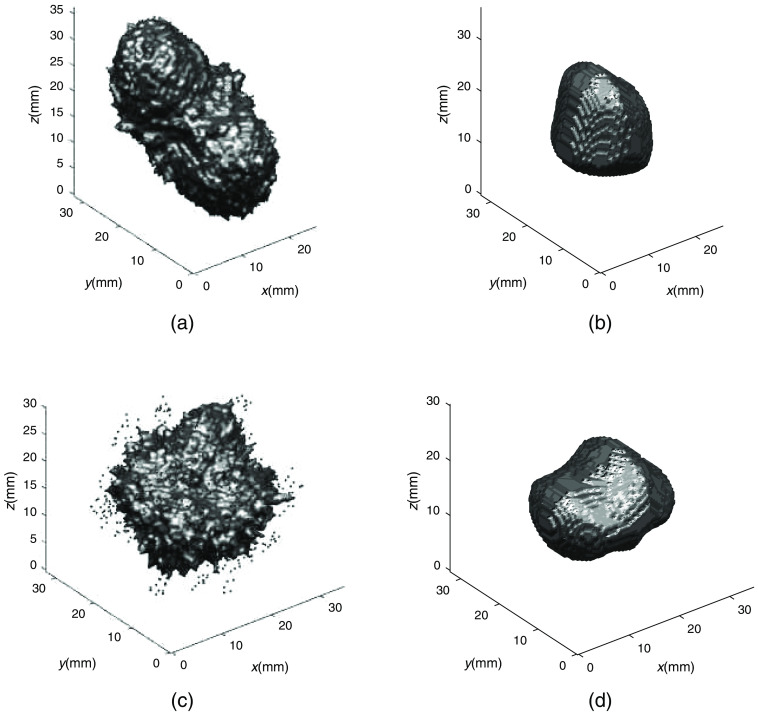
Ground truth and final extrapolation for example 1 from Fig. 4 and example 2 from Fig. 5. (a) Example 1: ground truth lesion. (b) Example 1: distance transform extrapolated shape, SDI=0.46, dV=−0.68. (c) Example 2: ground truth lesion. (d) Example 2: distance transform extrapolated shape, SDI=0.68, dV=−0.39.

In Figs. 4 and 5, we show some of the B-mode images simulated via the method presented in Sec. 2 and the main acoustic properties of the media. The images present characteristics of real B-mode images, such as shadowing and speckles. The method is able to simulate breast structures as they are present in the corresponding VICTRE digital phantom. The simulated inclusions result to be less scattering than the rest of the tissues in the background and their borders are highlighted by means of the simulated contrast in characteristic impedance with respect to the surrounding area. For each inclusion, a set of three to four control points was selected close to the internal border of the inclusion itself. An algorithm based on snake fitting^73^^,^^74^ retrieved a segmentation of the image as shown in Figs. 6(b) and 6(d). In Fig. 6, we show the results of the extrapolation routine described in Ref. 73. An assessment of the extrapolation procedure was performed by means of the Sørensen-Dice index (SDI):^75^^–^^77^

$$\mathrm{SDI}(X,Y)=\frac{2\underset{i}{\sum }|X_{i}\times Y_{i}|}{\underset{i}{\sum }|X_{i}|+\underset{i}{\sum }|Y_{i}|},$$ (17)

where $X_{i}$ and $Y_{i}$ are the i’th elements of two binary images X and Y ranging from 0 and 1, of the volume mismatch and of the displacement between the center of mass of ground truth and of the retrieved images. An analogous quantification has been performed for the segmentation procedure alone. Results are shown in Table 3. The SDI shows that the 3D extrapolation procedure introduces a source of error with respect to the previous step of segmentation. Other considered metrics were the relative mismatch in area dA and volume dV and the displacement of the center of the lesions. While the displacement of the inclusion results to be negligible with respect to the computational grid chosen for the optical reconstruction, there is a general underestimation of the size of the inclusions by about 50% of the ground truth value. This is mainly due to the different speed of sound of the inclusion with respect to the selected $v_{s,0}$ used in the image formation.

**Table 3:** Table displaying the performances of the segmentation and extrapolation procedure first presented in Ref. 73. In general, both the segmentation and the extrapolation procedure underestimate the dimensions of the ground truth shape. As expected, the SDI of the extrapolation procedure show a worse agreement between retrieved shape and ground truth with respect to the sole segmentation. Displacements in the lesion position negligible with respect to the grid resolution used here for DOT reconstruction.

	SDI	dA|dV	Displacement (mm)		
Segmentation	0.71(0.13)	−0.33(0.16)	Δx=0.03(0.13)	—	Δz=0.15(0.07)
Extrapolation	0.55(0.14)	−0.5(0.17)	Δx=0.04(0.13)	Δy=0.08(0.05)	Δz=0.14(0.08)

### 3.2 Assessment of Optical Reconstruction and Classification

**Fig. 7:**
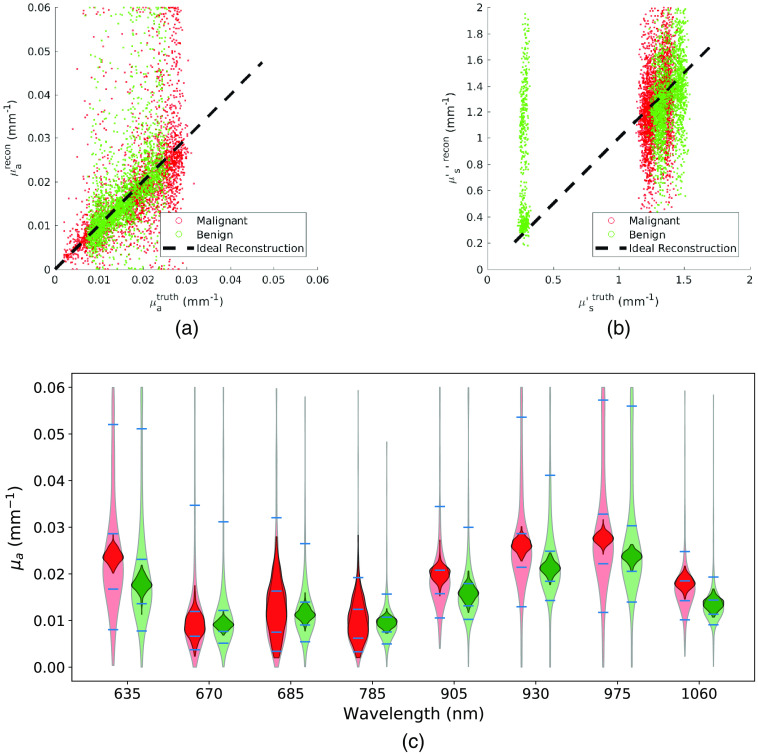
Comparison of ground truth optical coefficients and reconstructed ones using the ground truth shape as two-region delimiter. In (a) and (b), a scatter plot of reconstructed inclusions versus ground truth values are shown. Each scatter plot consists of 724  lesions×8  wavelengths scatter points. As can be seen, points tend to cluster around the optimal behavior highlighted by the black dashed line. As expected, this behavior is more accentuated for absorption. (a) Scatter plot of ground truth lesion absorption versus reconstructed lesion absorption. (b) Scatter plot of ground truth lesion scattering versus reconstructed lesion scattering. (c) Violin plot of ground truth (dark) and retrieved (light) absorptions by wavelength. Green represents benign lesions and red malignant ones. Blue ticks represent the 5th, 25th, 75th and 95th percentiles of the distribution of the retrieved absorptions. (c) Display of the statistics of the ground truth and retrieved values by wavelength.

A first assessment aimed at retrieving $\mu_{a}$ and ${\mu_{s}}^{\prime }$ inside the inclusion with the two regions defined by the ground truth lesion. In Fig. 7, we plot all the retrieved values of absorption and scattering coefficients obtained in reconstruction versus the ground truth value. The black dashed line in Fig. 7 shows the ideal results for which the reconstructed properties are identical to the ground truth. Especially with respect to absorption, a clustering of points along the bisector can be observed. This effect is still present but less prevalent for scattering. In Table 4, we show the average quantification errors for the lesions in the cases of two regions defined by the ground truth lesion shapes and defined by the US extrapolation procedure. As expected, the latter exhibits larger errors. Twenty-five percent of the retrieved values have a relative difference with the ground truth higher than 50%. The percentage decreases to 18% when the ground truth lesion shape is available. As a second step, reconstructions were performed by defining the two regions with the information retrieved from the US.

**Table 4:** Display of reconstruction error for the optical coefficients of the inclusion for a total of $N_{\mathrm{ph}}\times N_{\lambda }∼5600$ reconstructions. While the average error for both absorption is around limited to 13%, variability in the performances is higher as highlighted by the standard deviation in parenthesis. We also show for both coefficients the fraction of reconstructions with absolute error higher than 50%. The value of this is around 18% of reconstructions when using the ground truth lesions shape in the fit and increases to 25% when using US.

Two-region	$\varepsilon_{\mu_{a}}=\frac{\mu_{a}^{\text{recon}}-\mu_{a}^{\text{truth}}}{\mu_{a}^{\text{truth}}}$	$\frac{\#(|\varepsilon_{\mu_{a}}|>0.5)}{N_{\lambda }\times N_{\mathrm{ph}}}$	$\varepsilon_{{\mu_{s}}^{\prime }}=\frac{{\mu_{s}}^{\prime \text{recon}}-{\mu_{s}}^{\prime \text{truth}}}{{\mu_{s}}^{\prime \text{truth}}}$	$\frac{\#(|\varepsilon_{{\mu_{s}}^{\prime }}|>0.5)}{N_{\lambda }\times N_{\mathrm{ph}}}$
Ground	0.08 (0.64)	0.18	0.35 (0.79)	0.1
US	0.13 (0.76)	0.25	0.16 (0.89)	0.13

**Fig. 8:**
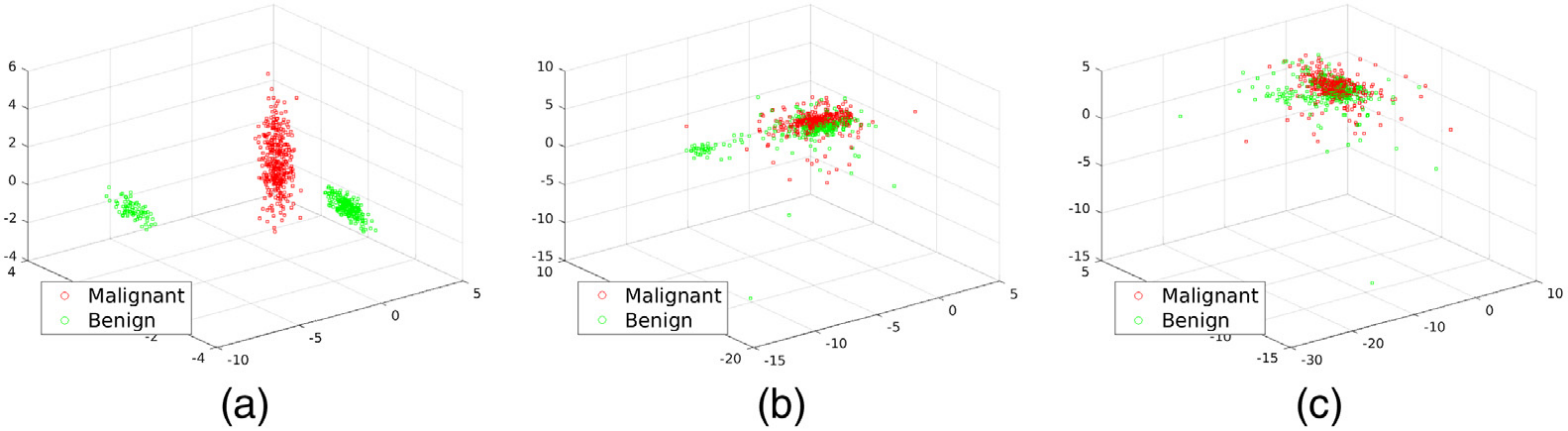
PCA of ground truth and reconstructed values of the inclusion with the three axes representing the first three principal components of the dataset. The separation is neat on the ground truth. A certain degree of separation can also be observed after reconstruction with the ground truth shape defining the two regions. Results are apparently worse when defining the two-region extrapolating a shape from the US B-mode simulations. However, the dataset can have better separability in higher dimensions or applying a nonlinear transformation. (a) PCA of ground truth—log normalization. (b) PCA of nonlinear model fit—log normalization. (c) PCA of nonlinear model fit with US prior—log normalization.

In Fig. 8, we show the scatter plot of the first three components for (i) ground truth, (ii) optical coefficients in the case of reconstruction with ideal two-region model, and (iii) reconstruction with US-extrapolated two-region. The separation of the phantoms into benign and malignant is clear for the ground truth. A noticeable decrease in separation is observed upon optical reconstruction from optical data, especially in the case of US-extrapolated two-region model.

Results of the application of logistic regression, SVM, and the FCN described in Sec. 2.3 are shown in Table 5. In general, the FCN performs better than other proposed methods, at a cost of larger training time (5 min) and fine tuning of the hyperparameters. All the applied methods show an optimal prediction for what concerns the ground truth. When the coefficients are retrieved with our reconstruction method, all the figures appreciably decrease. The accuracy, when the two regions are identified by the ground truth, is over 80%. In general, recall is higher than the precision, so malignant lesions are more easily identified as such with respect to benign ones.

**Table 5:** Overall results for classification. All considered methods can correctly classify all the lesions by giving their ground truth as input. Upon reconstruction with the ideal two-region definition, accuracy decreases to 83.5% in the best-case scenario, given by FCN. When the two-region definition is retrieved by the US image performances generally decrease. The best-case scenario is given by SVMs that reach an accuracy of 78%.

Model	Two-region	FCN (%)				SVM (%)				Log. Regr. (%)			
		Acc.	Prec.	Rec.	F1	Acc.	Prec.	Rec.	F1	Acc.	Prec.	Rec.	F1
Ground	—	100	100	100	100	100	100	100	100	100	100	100	100
Sep.Wav.	Ground	83.5	85.7	83.0	84.4	81.4	79.6	85.5	82.4	76.9	73.6	85.5	79.1
Sep.Wav.	US	75.4	76.1	79.7	77.9	78.0	79.3	76.7	78.0	69.1	68.5	72.5	70.4

## 4 Conclusion

We proposed a simulation pipeline based on VICTRE for the generation of realistic US B-mode images and DOT data. The method allows to generate a dataset for a newly designed probe on a shorter time scale and with larger variability than that which is obtainable experimentally in clinics. We generated a total of 349 benign and 379 malignant samples complete of B-mode images and optical data. We assessed a two-region fitting reconstruction method on the simulated data where the two-region system was extracted from the B-mode images. The procedure of segmentation and 3D prior extraction was evaluated. On average, the retrieved volumes resulted to underestimate the physical dimensions of the ground truth lesions of about 50% in volume. The displacement with respect to the center of mass of the lesions resulted to be less than the resolution of the computational grid of the optical reconstructions of 2 mm used here. The utilized optical reconstruction method does not suffer from model approximation error as the optical contrast between bulk and inclusion increases. In general, the reconstructed values of the inclusions approximate the actual values of the ground truth with somewhat frequent exceptions, absorption relative errors higher that 50% are present in 18% of reconstructions with an ideal two-region definition and 25% when the two regions are extracted from a US B-mode image. The number of reconstruction with relative errors higher than 50% generally increase when using also measurements coming from shorter source–detector distances in reconstruction, suggesting a limit of the two-region model. Nonetheless, a certain degree of separation between benign and malignant inclusions is still observable when doing principal component analysis on the log-normalized data. Three classification methods have been tested on the results of the reconstructions: an SVM, an FCN, and logistic regression. SVM and logistic regression show comparable results, while FCN has a better classification performances at the cost of a higher training time. The classification displays an accuracy close to 84% when using an ideal two-region reconstruction. When the two-region system is inferred by the US, a worsening in the performances of classification is observable, bringing the accuracy to 78%. Future steps will explore other methods for the extrapolation of the two regions from the US B-mode images. The availability of large datasets of lesion shapes and of corresponding US B-mode images allows to speculate the refinement of the extrapolation procedure with 3D shape reconstruction techniques utilizing supervised machine learning approaches.^78^ Other approaches for a possible improvement for the quantification of lesions optical properties may include the integration of a spectral model also in reconstruction as in Ref. 79. Future steps in the simulations may include more realistic ways to simulate the compression of breasts to make it better resemble the one given by handheld probes.

## Code, Data, and Materials availability

The code for the generation of digital phantoms and for optical and acoustic ground can be found at: https://github.com/giudisciacca/victre_codes. The code builds on the software suite VICTRE that can be found at: https://github.com/DIDSR/VICTRE. The code for the generation of US B-mode images requires the installation of K-wave and can be found at: https://github.com/giudisciacca/CLUSTER_COMPUTATION/tree/master/k-wave/myscripts/USfromVictre. The code for the segmentation of the images is at https://github.com/giudisciacca/CLUSTER_COMPUTATION/tree/master/k-wave/myscripts/USfromVictre, the generation of Diffuse Optical data and the can be found at: https://github.com/giudisciacca/CLUSTER_COMPUTATION/tree/master/SOLUS/example/VICTRE_SIMULATION_AND_RECON. It requires the installation of TOAST++ http://web4.cs.ucl.ac.uk/research/vis/toast/ and builds upon the software suite SOLUS: https://github.com/andreafarina/SOLUS. Codes for classification can be found in: https://github.com/giudisciacca/victre_codes/tree/main/pyClassify.
